# Non-invasive estimation of material properties of normal and dissected human ascending aortas *in vivo*: comparison with the *ex vivo* tensile experiment

**DOI:** 10.3389/fbioe.2025.1689692

**Published:** 2026-01-08

**Authors:** Xiaoya Guo, Yi Yang, Liang Wang, Dalin Tang, Haoliang Sun

**Affiliations:** 1 School of Science, Nanjing University of Posts and Telecommunications, Nanjing, China; 2 School of Biological Science and Medical Engineering, Southeast University, Nanjing, China; 3 Mathematical Sciences Department, Worcester Polytechnic Institute, Worcester, MA, United States; 4 Department of Cardiovascular Surgery, First Affiliated Hospital of Nanjing Medical University, Nanjing, China

**Keywords:** aortic dissection, ascending aortic tissue, *in vivo* material properties, finite element analysis, biaxial tensile test

## Abstract

**Objective:**

Patient-specific aortic material properties play a critical role in aortic dissection development. In this study, a non-invasive method was employed to assess the *in vivo* anisotropic mechanical properties of normal and dissected ascending aortas and compare them with their *ex vivo* material properties.

**Methods:**

Biaxial tensile testing was performed on 10 ascending aortic specimens (five patients with type-A aortic dissection and five donors without aortic diseases), with testing data fitted using anisotropic Mooney–Rivlin models. An iterative algorithm was proposed to determine *in vivo* aortic material properties by matching systolic and diastolic aortic geometries from echocardiography images with those from computed tomography-based computational models. Three settings of initial guesses of material parameters (M_01_: subject-specific *ex vivo* parameters; M_02_: *ex vivo* parameters of one subject with median stiffness; M_03_: a 5% variation applied to M_02_) were investigated in the iterative algorithm for their influence on *in vivo* property estimation and effective Young’s moduli along the circumferential (YMc) and axial (YMa) directions.

**Results:**

M_01_-derived *in vivo* properties had a maximum relative error of −33.44% in YMc/YMa among 10 subjects compared to *ex vivo* material properties. The median relative error of YMc was −29.40% for M_02_. Furthermore, a 5% variation in initial parameters caused less than 1.5% change in the estimated *in vivo* properties. The anisotropy difference between the initial material guess and real aortic tissue would exert a significant impact on YMa estimation but negligible effects on stress distributions.

**Conclusion:**

Overall, *in vivo* material properties estimated using the proposed method exhibited lower YMc values than the experimental results for normal and dissected ascending aortas.

## 1 Introduction

Aortic dissection (AD) is a life-threatening disease mainly characterized by the tearing of the aortic intima. The tear further extends, splitting the layers of the aortic wall and creating a false lumen (an abnormal, new channel of blood flow that forms in the aortic media), which in turn could lead to blood flow deficiency and eventually mortality (Nienaber et al., 2016). Stanford type-A AD involves the ascending aorta and typically manifests as sudden, severe chest pain, necessitating emergency surgical interventions to repair or replace the affected aortic segment (Ma et al., 2013; El Hussein and Green, 2022). Imaging modalities, such as computed tomography (CT), magnetic resonance imaging (MRI), transthoracic echocardiography (TTE), and transesophageal echocardiography (TEE), are employed for diagnosing the disease in clinics. When TEE examinations could not yield conclusive results, further investigation with CT or MRI is recommended (Flachskampf, 2006). The diameter of the ascending aorta measured in medical imaging was used as the reference basis (≥5.5 cm) for prophylactic ascending aorta replacement (Erbel et al., 2014). However, approximately 40% of ascending aortic dissections occur below this threshold (Pape et al., 2007). Therefore, a better understanding of aortic biomechanics could facilitate the development of more effective criteria for enabling accurate prediction (Raghavan et al., 1996; Chung et al., 2014; Korenczuk et al., 2019).

Mechanical properties of the aortic wall play a crucial role in determining the biomechanical behavior of the aorta. Therefore, advancing the mechanical characterization of the aorta is critical to improving biomechanical analyses for AD. Studies comparing healthy and aneurysmal human ascending aortas through *ex vivo* tensile experiments have demonstrated significantly increased stiffness in pathological tissues (Choudhury et al., 2009; Azadani et al., 2013; Emmott et al., 2016; Durmaz, 2023). Azadani et al. (2013) used biaxial stretch testing to evaluate mechanical properties and indicated significant differences in physiological stresses between healthy and aneurysmal tissues in both circumferential (YMc) (3,041.4 ± 1,673.7 vs. 905.1 ± 358.9 kPa; p < 0.001) and axial (YMa) (3,498.2 ± 2,456.8 vs. 915.3 ± 368.9 kPa; p < 0.001) directions. Kruger et al. (2016) performed uniaxial tensile testing to assess directional tissue compliance and failure metrics, suggesting that a high axial compliance and low failure stress of the ascending aorta may cause predisposition to recurrent patho-anatomy of type-A AD. A recent study comparing dilated and normal aortic tissues revealed that aneurysmal tissues were much stiffer, although no significant histopathological changes were detected (Durmaz, 2023).

The *ex vivo* experiments revealed associations between mechanical properties and pathological changes in the ascending aorta, implying that biomechanical analyses may contribute to predicting disease progression. However, one major barrier to performing the biomechanical analysis of ascending aortas is the difficulty in acquiring patient-specific material properties (Wang et al., 2022). The aforementioned tensile experiments could only be performed on excised tissues *ex vivo* and are not suitable for evaluating the aortic mechanical properties *in vivo*. Fortunately, considerable advancements in medical imaging and computational modeling show the potential for determining patient-specific aortic material properties *in vivo*. Prior studies utilized patient-specific blood pressures and TEE images for better capturing the *in vivo* mechanical behaviors of ascending aortas and reported that TEE-derived stiffness had a significant correlation with *ex vivo* aortic wall biomechanics (Alreshidan et al., 2017; Emmott et al., 2018). Deplano et al. (2019) first performed biaxial tensile testing on the dissected intimomedial flap and *in vivo* stiffness measurements for patients with acute type-A dissection. Cardiac MRI, non-invasive TTE, and CT can also be used for evaluating aortic elastic properties and even aortic biomechanical stress states to explore their link with the pathogenesis of dissection disease (Cosentino et al., 2019; Morgant et al., 2021; Fortunato et al., 2022).

In this study, a non-invasive method was employed to estimate the *in vivo* material properties of normal and dissected human ascending aortas. To this purpose, a finite element model-based iterative algorithm was proposed to evaluate the subject-specific aortic material properties *in vivo* by matching the aortic movement on TEE images for five patients with type-A dissection and five donors without aortic diseases. Subsequently, the results were compared with material properties from *ex vivo* biaxial experiments conducted on the ascending aortic samples harvested from these subjects. Finally, the influence of the initial guesses of material parameters on *in vivo* material property estimation and stress distributions was analyzed.

## 2 Materials and methods

### 2.1 Acquisition of clinical information and aortic tissue specimens

Medical images and ascending aortic specimens were collected from five patients with type-A AD (AD group, denoted as AD1 to AD5) who underwent ascending aortic resection. Patients with connective tissue disease such as Marfan syndrome or connective tissue disorders and iatrogenic or traumatic dissection were excluded from the cohort. Patients who could not undergo TEE and CT examinations were also excluded. All ascending aortic specimens were obtained intraoperatively from Jiangsu Province Hospital with informed consent obtained from all participating patients. The preoperative TEE and CT examinations were performed in a GE Vivid E95 machine with the 6Vt-D probe (GE HealthCare, Madison, WI) and CT scanner (SOMATOM Definition AS, Siemens AG), respectively. Arm blood pressures were also recorded. For comparison purposes, normal ascending aorta tissues from five organ donors without aortic diseases (N group, denoted as N1 to N5) were also collected. Since no imaging examinations were performed for these five donors, CT and TEE images from five matched volunteers with no aortic diseases were used as substitutes for these five subjects (matching information included age, gender, and pressure conditions). The clinical information, image data, and tissue samples were acquired following the protocol approved by the Medical Ethics Committee of Jiangsu Province Hospital (approval number: 2022-SR-730). In total, 10 aortic tissue specimens were harvested, with one specimen from each subject for mechanical testing, and the corresponding TEE and CT images and blood pressure data were recorded to construct finite element models.

### 2.2 Biaxial tensile testing

**FIGURE 1:**
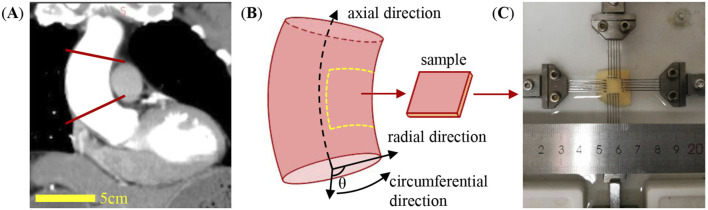
Ascending aortic tissue sample characterization. **(A)** CT image of the ascending aorta from a patient in the longitudinal view. **(B)** Diagram showing the axial, radial, and circumferential directions of the aortic tissue and the testing sample. **(C)** Biaxial tensile testing of the aortic sample.

After excision of ascending aortas during operation, tissue specimens were stored at −80 °C in a cryopreservation solution until subsequent use for biaxial tensile testing (Martin et al., 2011; Kural et al., 2012; Teng et al., 2014). The surrounding adipose tissue was removed from the surface of the aortic adventitia after defrosting. Square samples with the dimension of approximately 2 cm × 2 cm were cut with the edges of the samples aligned along the circumferential and axial directions of the aortic tissues. Samples were excised from the non-dissected site adjacent to the dissection flap for all aortic specimens, which was located near the minor region (see Figure 1) (Choudhury et al., 2009). The thickness of each sample was determined by averaging four measurements taken within the gauge region using a digital caliper (Mitutoyo 500-197-30, resolution: 0.01 mm). Care was taken to ensure gentle contact during measurement to minimize tissue compression.

The samples were mounted on a biaxial tensile testing device (IPBF-300, CARE Measurement and Control Co., Ltd.) and immersed in phosphate-buffered saline at a temperature of approximately 37 °C. Ten cycles of preconditioning were performed to reduce tissue hysteresis. Each sample was then tested by conducting five consecutive loading protocols: force ratios (circumferential direction to axial direction) of 1:1, 1:0.75, 0.75:1, 1:0.5, and 0.5:1 with the maximal force of 2.0 N (Pena et al., 2015; Guo et al., 2023).

### 2.3 Constitutive modeling of ascending aortas

The anisotropic Mooney–Rivlin material model was selected to evaluate the mechanical properties of ascending aortas with the assumption of material homogeneity and incompressibility. The strain–energy function per unit reference volume of the modified anisotropic Mooney–Rivlin model (Holzapfel, 2000) is calculated as Equation 1.

$$W=c_{1}\left(I_{1}-3\right)+D_{1}\left(e^{D_{2}\left(I_{1}-3\right)}-1\right)+W_{ansio},W_{ansio}=\frac{K_{1}}{{2K}_{2}}\left(e^{K_{2}{\left(J_{4}-1\right)}^{2}}+e^{K_{2}{\left(J_{6}-1\right)}^{2}}-2\right),$$ (1)

where $I_{1}=\sum C_{ij}\text{ and }I_{2}=\frac{1}{2}\left({I_{1}}^{2}-C_{ij}C_{ij}\right).$ $I_{1}$ and $I_{2}$ are the first and second invariants of right Cauchy–Green deformation tensor $C=\left[C_{ij}\right]=X^{T}X,\text{where }X=\left[X_{ij}\right]=\left[\partial x_{i}/\partial a_{j}\right]$ and $\left(x_{i}\right)$ and $\left(a_{j}\right)$ are the current and original positions, respectively. $J_{4}=I_{4}{I_{3}}^{-\frac{1}{3}}\text{ and }J_{6}=I_{6}{I_{3}}^{-\frac{1}{3}},{where\;I}_{4}=C_{ij}{\left(n_{a}\right)}_{i}{\left(n_{a}\right)}_{j},I_{6}=C_{ij}{\left(n_{b}\right)}_{i}{\left(n_{b}\right)}_{j},$ and $I_{3}$ is the third invariant of C . $n_{a}$ and $n_{b}$ represent fiber directions, which were assumed to be symmetric about the circumferential direction (Holzapfel, 2006), with the angle between the fiber orientation and the circumferential direction denoted by *θ*. $c_{1},D_{1},D_{2},K_{1},$ and $K_{2}$ denote material parameters, which were constrained to be positive. Niestrawska et al. (2016) reported that the median angle between the mean fiber direction and the circumferential direction in aortic tissues was 24.46° (Q1–Q3: 22.45°–30.18°) based on second-harmonic-generation images; thus, the parameter *θ* was constrained to the range (0°, 45°) for material parameter fitting. The material parameters were optimized using the trust-region-reflective algorithm (Moré and Sorensen, 1983). The coefficient of determination (*R* ^*2*^) was calculated to measure the goodness-of-fit of parameter fitting.

### 2.4 Medical image processing

**FIGURE 2:**
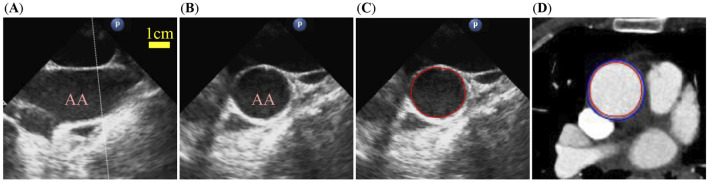
Medical images and segmentations of the ascending aorta. **(A)** Long-axis view of the TEE image. **(B)** Short-axis view of the TEE image at the position of the white dotted line in **(A)**. AA, ascending aorta. **(C)** Perimeter of the lumen marked in the TEE image. **(D)** Lumen and wall contours marked in the CT image.

CT and TEE images of each patient were selected for segmentation to allow for quantification of the ascending aorta’s morphology at the exact location where the tissue samples were harvested (Figure 2). The locations typically are approximately 1–5 cm above the sinotubular junction of the aorta. Segmented CT images provided the lumen and outer-boundary contours of aortas. Then the thickness of the aorta specimens measured *ex vivo* was used as the thickness threshold to modify the segmented outer-boundary contours as the segmentation accuracy may be affected by the surrounding adipose tissue adhered to the surface of the aortic adventitia (Guo et al., 2023). More details are provided in Supplementary File Section A. The aortic deformation over one cardiac cycle was recorded through a sequence of TEE images. The level-set method was performed to segment the lumen contours in all TEE images (Yezzi et al., 1997). The dynamic change in the lumen circumference of the ascending aorta over one cardiac cycle was quantified using lumen contours from TEE images to determine the parameters of material models.

### 2.5 Image-based thin-slice models

In our computational models, the governing equations (motion equation, strain–displacement equation, and constitutive equation) described using the summation convention, are given by Equations 2–4, as follows (Huang et al., 2016):

$$\rho \frac{\partial^{2}v_{i}}{\partial t^{2}}=\frac{\partial \sigma_{ij}}{\partial x_{j}},i=1,2,3;$$ (2)

$$\varepsilon_{ij}=\frac{1}{2}\left(\frac{\partial v_{j}}{\partial a_{i}}+\frac{\partial v_{i}}{\partial a_{j}}+\frac{\partial v_{\alpha }}{\partial a_{i}}\frac{\partial v_{\alpha }}{\partial a_{j}}\right),i,j=1,2,3;$$ (3)

$$\sigma_{ij}=\frac{1}{2}\left(\frac{\partial W}{\partial \varepsilon_{ij}}+\frac{\partial W}{\partial \varepsilon_{ji}}\right),$$ (4)

where σ is the stress tensor, ε is the Green strain tensor, ρ is the density, x is the current position, a is the original position, v is the displacement (i.e., x−a ), and W is the strain–energy density function of the modified anisotropic Mooney–Rivlin model.

**FIGURE 3:**
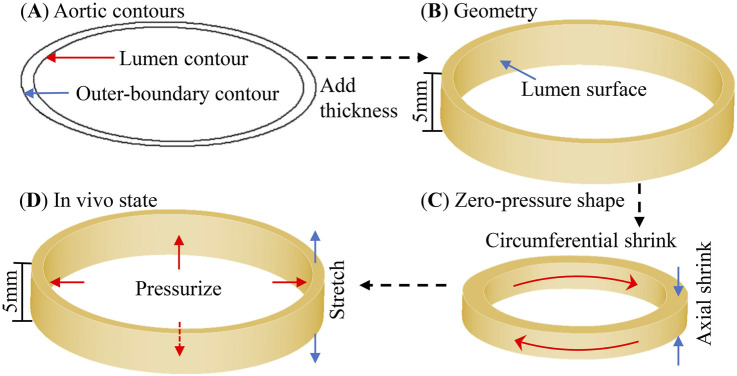
Modeling process of the 3D thin-slice model using aortic contours obtained from medical images. **(A)** Aortic contours. **(B)** Reconstructed 3D geometry. **(C)** Model in the zero-pressure reference state. **(D)** Model in the *in vivo* loaded state.

The 3D thin-slice model (Figure 3B) was constructed by adding a 5 mm thickness (CT slice thicknesses) onto 2D slices to apply axial shrinkage and stretch in the model. A critical challenge in constructing 3D models based on *in vivo* data lies in determining the axial and circumferential shrinkage rates required to obtain the zero-pressure geometry (used as the numerical initial state) (Figure 3C) while ensuring restoration of its *in vivo* geometry (Figures 3B,D) under physiological pressure and axial stretch conditions (Krishnan et al., 2015). Based on the established range of aortic axial stretch [1.1, 1.4] reported in prior studies (Desyatova et al., 2020; Guo et al., 2023), an average value of 1.25 was adopted in our model, corresponding to an axial shrinkage of 20%. The circumferential shrinkage rate and *in vivo* material parameters were determined using the proposed iterative algorithm detailed in the next section. Patient-specific pulsatile pressure conditions were applied to the lumen surface of 3D thin-slice models. These simulations were solved using ADINA software (Adina R & D, Watertown, MA, United States). Mesh analysis was performed by increasing mesh density by 10% in iterative steps and continued until the relative changes in the solutions was less than 1% (Guo et al., 2017; Guo et al., 2022). After achieving the stability of the solution through simulation of three cardiac cycles (using a time-step of 0.01 s), the stress field at the time of maximum pressure in the final cycle was extracted for subsequent evaluation, as the aortic tissue experienced the maximum external loading at this instant and consequently exhibited the highest stress levels.

### 2.6 The iterative algorithm to determine *in vivo* material parameters

**FIGURE 4:**
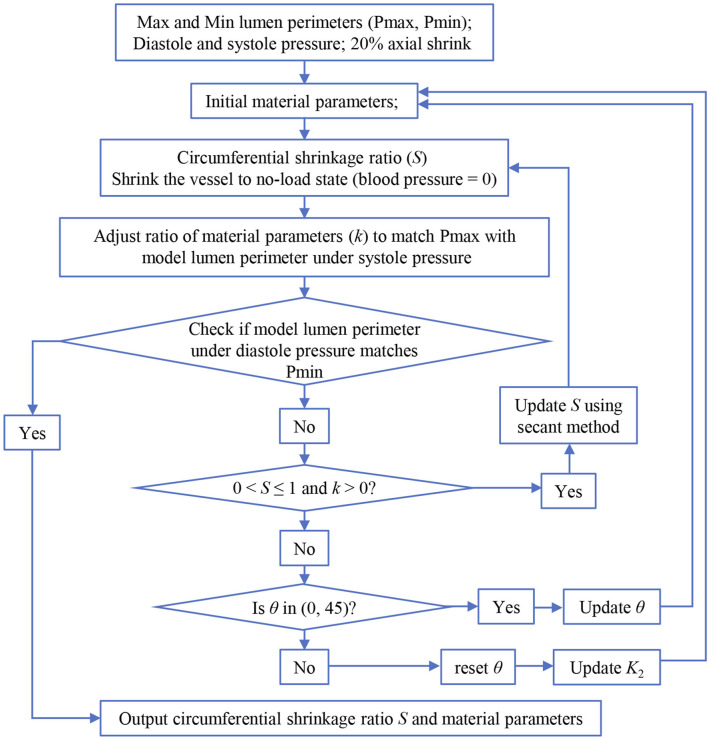
Flow chart of the iterative algorithm for determining *in vivo* material parameters.

To determine *in vivo* material properties of the ascending aorta for each patient-specific thin-slice model, a triple-loop iterative algorithm was employed to perform successive corrections of parameter values of the anisotropic Mooney–Rivlin model and to align the circumferential shrinkage with the maximum and minimum aortic lumen circumferences corresponding to systolic and diastolic pressures, respectively (Figure 4). As there are only two conditions to compare in our iterative algorithm, only two quantities can be determined in the inner loop, namely, circumferential shrinkage ratio (denoted as *S*) and the ratio of material parameters (denoted as *k*) (Guo et al., 2017). In the inner loop, parameters *K*_2_ and *θ* were kept constant, while other parameters of the initial guess were proportionally adjusted to correspond to the ratio *k*. In fact, determining the *in vivo* anisotropic material parameters is a complex multi-parameter nonlinear inverse problem. The final solution derived from this inverse approach is heavily influenced by the initial guess of material parameters. When the initial guess of material parameters results in a large deviation (especially the degree of anisotropy) from the actual material tissue properties, the obtained solution fails to satisfy the constraint conditions (i.e., the reasonable physiological conditions), denoted as 0 < *S* ≤ 1 and *k* > 0. In this case, initial material parameters *K*_2_ and *θ* in the anisotropic term would undergo successive corrections in the middle and outer loops until an optimal solution is reached. Here, the step sizes for *K*_2_ and *θ* were set to 0.01 and 1°, respectively. Finally, the circumferential shrinkage ratio *S* and the determined *in vivo* material parameters were obtained as outputs.

To study the effect of initial guesses of material parameters on the model outputs, three settings of initial material parameters were investigated following the *in vivo* method described above. Setting 1: subject-specific *ex vivo* material parameters were selected as the initial guess of material parameters (denoted as M_01_) for the corresponding subject. Setting 2: to set a common initial guess for all subjects for application purposes, *ex vivo* material parameters of patient AD1 were selected as the initial guess of material parameters for all models (denoted as M_02_): *c*_1_ = 2.6970 kPa; *D*_1_ = 15.1322 kPa; *D*_2_ = 0.9272; *K*_1_ = 2.1722 kPa; *K*_2_ = 0.9338; and *θ* = 5.56°, assuming that the aortic stiffness of patient AD1 is close to the median among 10 subjects. Setting 3: to analyze any possible uncertain effect on the initial value selection, a 5% variation was applied to each material parameter of patient AD1 (denoted as M_03_): *c*_1_ = 2.6373 kPa; *D*_1_ = 15.2031 kPa; *D*_2_ = 0.9696; *K*_1_ = 2.2732 kPa; *K*_2_ = 0.9018; and *θ* = 5.82°. Using the three settings of initial material parameters, M_01_, M_02_, and M_03_, the *in vivo* material parameters determined using the iterative algorithm were denoted as M1, M2, and M3, respectively.

### 2.7 Data analysis

To compare tissue stiffness across samples and between *in vivo* and *ex vivo* conditions, the effective Young’s modulus was determined from the slope of the linear proportional function fitted to the stress–stretch curves over the stretch interval [1.0, 1.3] (Markodimitrakis et al., 2023). The effective Young’s moduli along the circumferential and axial directions were calculated and denoted as YMc and YMa, respectively. Furthermore, material anisotropy index (AI) was calculated using Equation 5 with the effective Young’s modulus as the biomechanical variable for comparison (Chung et al., 2017):

$${\text{AI}}_{\text{YM}}=\frac{\text{YMc}-\text{YMa}}{\frac{1}{2}\left(\text{YMc}+\text{YMa}\right)}.$$ (5)

As variables did not meet the normality assumption based on the Shapiro–Wilk test, all continuous data were reported as the median with the interquartile range. The comparative analysis between *in vivo* and *ex vivo* measurements was performed using the Wilcoxon signed-rank test with a one-sided alternative hypothesis. Statistical analysis was performed using Python with a statistical significance level of 0.05.

## 3 Results

### 3.1 Patient information and geometric measurements

Table 1 summarizes clinical demographics and aortic characteristics for five patients with AD and five donors with a normal aorta, including age, sex, cuff pressure, sample thickness, and maximum and minimum lumen perimeters extracted from TEE images during one cardiac cycle.

**TABLE 1:** Patient information and measurements.

Patient no.	Age (years)	Sex (F/M)	Cuff pressure (mmHg)	Thickness (mm)	Minimum perimeter (cm)	Maximum perimeter (cm)	Change in perimeter
AD1	48	M	133/76	2.42	12.62	13.88	9.98%
AD2	57	M	119/65	1.74	13.43	14.48	7.82%
AD3	61	M	136/91	2.51	11.10	11.72	5.59%
AD4	58	F	140/91	2.80	9.60	9.89	3.02%
AD5	55	M	113/60	1.29	10.58	12.96	22.5%
N1	37	M	88/47	1.66	8.50	9.69	14.00%
N2	32	M	117/61	1.33	9.19	10.58	15.13%
N3	42	M	79/45	1.31	9.16	10.33	12.77%
N4	45	F	111/61	1.44	9.18	9.97	8.61%
N5	54	M	97/59	1.20	9.94	10.89	9.56%

### 3.2 *Ex vivo* material properties using biaxial tensile testing

Stress–strain data derived from biaxial tensile experiments were used to determine the material parameters of the anisotropic Mooney–Rivlin model for 10 samples. The adopted material model demonstrated a good fit for all samples, with *R* ^*2*^ values exceeding 0.9188 (see Supplementary Figures S1 and S2 in Supplementary File for the material curve fits of all samples). The material parameters of the anisotropic Mooney–Rivlin model and *R* ^*2*^ values obtained by fitting the *ex vivo* experimental data are shown in Table 2.

**TABLE 2:** Material parameters of the Mooney–Rivlin model obtained from *ex vivo* experiments.

Patient no.	*c*_1_ (kPa)	*D*_1_ (kPa)	*D*_2_	*K*_1_ (kPa)	*K*_2_	*θ* (°)	*R* ^*2*^
AD1	2.6970	15.1322	0.9272	2.1722	0.9338	5.56	0.9898
AD2	0.4730	12.0210	0.9184	0.2379	1.3761	12.43	0.9684
AD3	0.6100	6.2073	2.2917	1.5853	10.2653	8.07	0.9795
AD4	0.5100	6.2066	2.1138	0.8650	15.1746	7.79	0.9629
AD5	0.9800	22.9048	0.8946	0.0100	0.0105	40.01	0.9188
N1	0.0101	29.3547	0.5745	1.3323	1.0643	5.80	0.9903
N2	1.0000	14.4936	1.4818	0.1000	0.5000	39.56	0.9478
N3	0.5400	12.1538	1.5536	0.0100	0.0100	14.95	0.9211
N4	1.6478	17.6785	1.0153	0.3353	2.7247	12.00	0.9829
N5	18.3579	5.1581	1.9418	1.1775	4.5227	9.60	0.9905

### 3.3 Comparison of *in vivo* and *ex vivo* material properties

**FIGURE 5:**
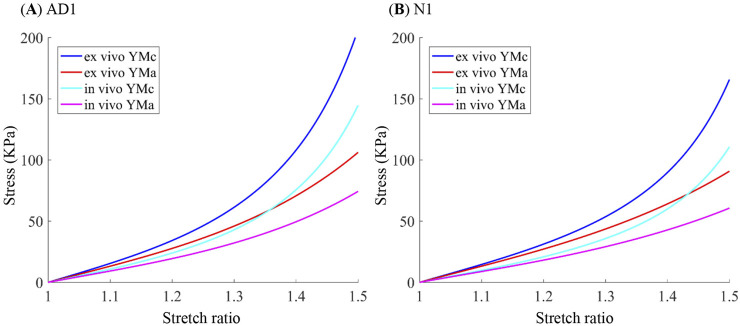
Comparison of stress–strain curves for AD1 and N1 based on the anisotropic Mooney–Rivlin model using *ex vivo* and *in vivo* material parameters. **(A)** Patient AD1. **(B)** Donor N1.

Setting 1: To compare the differences between *ex vivo* material properties obtained from biaxial tensile testing and *in vivo* material properties determined using the iterative algorithm, the *ex vivo* material parameters (Table 2) were assigned as the initial parameters (M_01_) in the material model for each corresponding patient or donor. The triple-loop algorithm achieved convergence in the inner loop, with the circumferential shrinkage ratio *S* and the ratio of material parameters *k* for 10 models summarized in Table 3. The effective Young’s moduli (YMc and YMa) of both *in vivo* and *ex vivo* material properties were calculated for comparison. The *in vivo* iterative algorithm resulted in softer material properties in both circumferential and axial directions than the corresponding *ex vivo* aortic tissue except for AD3 and AD4. Wilcoxon signed-rank tests indicated that the *in vivo* algorithm yielded significantly lower YMc (W = 0.0, p = 0.001, and r = 0.98) and YMa (W = 8.0, p = 0.024, and r = 0.62) than the *ex vivo* material. Using the *ex vivo* material properties as the baseline, the maximum relative error (RE) of YMc and YMa was 33.44% for the *in vivo* material results among 10 subjects. The median REs for YMc and YMa among 10 subjects were −14.50% (Q1 = −19.66%; Q3 = −6.67%) and −8.54% (Q1 = −17.31%; Q3 = −4.66%), respectively. Figure 5 shows the stress–strain curves together with the *ex vivo* testing data.

**TABLE 3:** Comparison of the stiffness of the *in vivo* material properties determined by the initial guess M_01_ and *ex vivo* material properties.

Patient no.	*In* *vivo*					*Ex* *vivo*				
	*S* (%)	*k*	YMc (kPa)	YMa (kPa)	AI_YM_	YMc (kPa)	YMa (kPa)	AI_YM_	YMc RE (%)	YMa RE (%)
AD1	68.27	0.70	126.38	100.05	0.23	181.46	142.92	0.24	−30.35	−30.00
AD2	63.32	0.93	96.99	93.06	0.04	104.90	100.06	0.05	−7.54	−7.00
AD3	81.71	0.98	803.96	161.31	1.33	960.24	160.14	1.43	−16.28	0.73
AD4	82.52	1.22	3247.44	179.87	1.79	3413.61	142.87	1.84	−4.87	25.90
AD5	85.38	0.90	167.17	167.15	0.00	178.56	178.51	0.00	−6.38	−6.37
N1	57.76	0.67	108.67	92.67	0.16	163.27	138.73	0.16	−33.44	−33.20
N2	70.28	0.81	174.49	174.27	0.00	208.60	208.35	0.00	−16.35	−16.35
N3	80.98	0.96	181.07	180.96	0.00	219.94	219.68	0.00	−20.76	−17.63
N4	71.51	0.96	173.46	164.92	0.05	182.46	171.96	0.06	−4.93	−4.09
N5	82.41	0.90	270.60	217.57	0.22	310.02	241.95	0.25	−12.71	−10.08

Setting 2 and Setting 3: The *in vivo* material parameters obtained using the iterative algorithm based on the initial material settings M_02_ and M_03_ are listed in Tables 4 and 5, respectively. For initial conditions M_02_ and M_03_, convergence was achieved at the inner (n = 6, 7), middle (n = 3, 2), and outer (n = 1, 1) loops, respectively. This evaluation demonstrates that this algorithm can be applied to yield a convergent solution across a wide range of initial guesses of the material parameters. Compared with *ex vivo* material properties, the median REs of YMc determined under M_02_ and M_03_ for 10 subjects were −29.40% (Q1 = −30.73%; Q3 = −21.26%) and −27.74% (Q1 = −30.09%; Q3 = −20.88%), respectively. The median REs of YMa determined under M_02_ and M_03_ for 10 subjects were −28.51% (Q1 = −34.83%; Q3 = −17.47%) and −31.00% (Q1 = −34.93%; Q3 = −18.41%), respectively. Wilcoxon signed-rank tests indicated that the YMc values obtained from *in vivo* experiments under M_02_ (W = 1.0, p = 0.002, and r = 0.913) and M_03_ (W = 0.0, p = 0.001, and r = 0.979) were significantly lower than those obtained from *ex vivo* experiments.

**TABLE 4:** *In vivo* material parameters of the Mooney–Rivlin model obtained from the iterative algorithm using the *ex vivo* material parameters of AD1 as the initial material parameters (M_02_).

Patient no.	*c*_1_ (kPa)	*D*_1_ (kPa)	*K*_1_ (kPa)	*K*_2_	*θ* (°)	AI_YM_	YMc (kPa)	YMa (kPa)	YMc RE (%)	YMa RE (%)
AD1	1.8879	10.5925	1.5206	0.9338	5.56	0.23	126.38	100.05	−30.35	−30.00
AD2	1.5950	8.9489	1.2846	0.9338	5.56	0.23	106.77	84.52	1.79	−15.53
AD3	3.3416	18.7487	2.6914	7.9338	5.56	1.18	683.32	177.08	−28.84	10.58
AD4	36.4397	204.4523	29.3495	1.9338	5.56	0.30	2621.01	1931.02	−23.22	1251.57
AD5	2.4893	13.9670	2.0050	0.9338	40.56	0.03	141.75	136.97	−20.61	−23.27
N1	1.7140	9.6165	1.3805	0.9338	5.56	0.23	114.37	90.54	−29.95	−34.74
N2	2.8696	16.1006	2.3113	0.9338	5.56	0.23	192.10	152.07	−7.91	−27.01
N3	2.3830	13.3705	1.9194	0.9338	5.56	0.23	159.53	126.29	−30.86	−42.51
N4	1.8178	10.1991	1.4641	0.9338	5.56	0.23	121.68	96.33	−33.31	−43.98
N5	2.9743	16.6877	2.3956	1.9338	5.56	0.30	213.93	157.61	−30.99	−34.86

D_2_ was fixed at 0.9272 for all 10 subjects.

**TABLE 5:** *In vivo* material parameters of the Mooney–Rivlin model obtained from the iterative algorithm using the altered initial material parameters (M_03_).

Patient no.	*c*_1_ (kPa)	*D*_1_ (kPa)	*K*_1_ (kPa)	*K*_2_	*θ* (°)	AI_YM_	YMc (kPa)	YMa (kPa)	YMc RE (%)	YMa RE (%)
AD1	1.6615	9.5780	1.4321	0.9018	5.82	0.23	118.82	94.31	−34.52	−34.01
AD2	1.4505	8.3617	1.2503	0.9018	5.82	0.23	103.73	82.33	−1.11	−17.72
AD3	3.1747	18.3015	2.7365	8.2018	5.82	1.22	745.23	180.20	−22.39	12.53
AD4	34.7756	200.4725	29.9748	0.9018	5.82	0.23	2486.87	1973.87	−27.15	1281.57
AD5	2.3735	13.6828	2.0459	0.9018	44.82	0.00	142.16	141.96	−20.38	−20.47
N1	1.5831	9.1264	1.3646	0.9018	5.82	0.23	113.21	89.86	−30.66	−35.23
N2	2.5951	14.9599	2.2368	0.9018	5.82	0.23	185.58	147.30	−11.04	−29.30
N3	2.2042	12.7068	1.8999	0.9018	5.82	0.23	157.63	125.11	−28.33	−43.05
N4	1.6335	9.4168	1.4080	0.9018	5.82	0.23	116.82	92.72	−35.98	−46.08
N5	2.8693	16.5410	2.4732	2.0018	5.82	0.31	222.09	162.86	−28.36	−32.69

D_2_ was fixed at 0.9696 for all 10 subjects.

To further validate our observation that the discrepancies in the initial guess for anisotropy lead to the occurrence of this outlier in the YMa from AD4, a sensitivity analysis was performed to study the impact of different initial anisotropy on the relative error in YMa. As parameter K_1_ primarily controls tissue anisotropy, its value was gradually increased from K_1_ = 2.1722 kPa (the initial guess in M_02_) to 80 times the baseline K_1_ using an incremental step of 20 times the baseline K_1_, while other material parameters were kept constant. As a result, the anisotropy index of the last case (K_1_ = 80*2.1722 kPa in Table 6) was comparable to that of AD4 (AI_YM_ = 1.84). The estimated *in vivo* material properties for all cases are provided in Table 6. It clearly shows that an increase in the K_1_ value (tissue anisotropy comparable to *ex vivo* aortic tissue of AD4) leads to a reduction of −10.77% in YMa RE from the initial 1,251.57% using M_02_ as the initial guess.

**TABLE 6:** *In vivo* material properties with variation in the initial *K*_1_ coefficient.

*K*_1_ (kPa)	AI_YM_	YMc (kPa)	YMa (kPa)	YMc RE (%)	YMa RE (%)
2.1722 (baseline)	0.30	2621.01	1931.02	−23.22	1251.57
20 × 2.1722	1.45	3016.46	481.67	−11.63	237.14
40 × 2.1722	1.68	2923.99	253.79	−14.34	77.64
60 × 2.1722	1.77	2913.70	173.68	−14.64	21.56
80 × 2.1722 (AI_YM_ close to AD4)	1.83	2808.02	127.48	−17.74	−10.77

The aforementioned explanation demonstrates that the large RE in YMa for AD4 was due to the deviation in the initial guess of anisotropy. To further explore why the initial guess M_02_ (with middle aortic stiffness among all 10 subjects) varied significantly from the *ex vivo* material properties of AD4, we examined the tissue microstructure of aortic tissue from AD4 using hematoxylin and eosin (H&E) staining and Oil Red O staining. Gross observation showed that aortic tissue from AD4 exhibited obvious atherosclerotic features and more disorganized tissue fiber orientation compared to other tissue samples (Supplementary Figure S3). This microstructural alteration might have contributed to the change in the material anisotropy, thus leading to the high RE value in YMa for AD4.

### 3.4 Impact of the initial parameter settings on aortic biomechanical stress conditions

**FIGURE 6:**
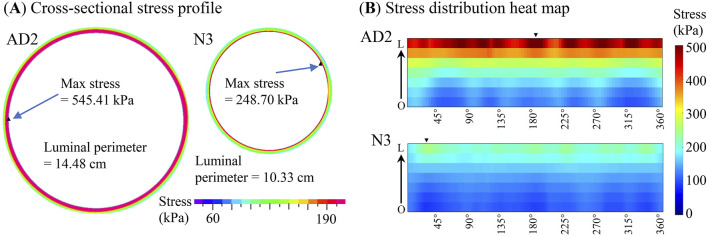
Stress distributions under systolic pressure conditions using material parameters M2 for AD2 and N3. **(A)** Cross-sectional stress profile, with the cross section uncoiled (starting from the rightmost point and proceeding counterclockwise) to reveal the stress distribution. **(B)** Corresponding heat maps of stress distributions. The direction from the outer boundary (O) to the lumen (L) is indicated by a black arrow, and the sites of maximum stress are marked by black triangles.

As an important biomechanical parameter, aortic wall stress influences AD progression. It is worth exploring how these *in vivo* material properties influence the aortic stress distributions. Three initial material settings (M_01_, M_02_, and M_03_) were applied to each subject, yielding three corresponding sets of *in vivo* material parameters (M1, M2, and M3). These material parameter sets were subsequently utilized to construct thin-slice models for aortic stress distribution simulations. Figure 6 shows the stress distributions under systolic pressure predicted by the finite element models of a sample patient and a sample control. The maximum principal stress on the lumen surface under systolic pressure was extracted for subsequent analysis, termed stress for convenience. The maximum stress value of each model is provided in Table 7. Using the stress derived from material parameters M1 as the reference, the maximum RE in stress calculations using M2 and M3 was 13.84% among the 10 patients. In these 10 subject-specific models, a 5% variation in initial material parameters caused less than 1.5% change in the stress value.

**TABLE 7:** Maximum stress on the aortic lumen surface under systolic pressure derived from three different groups of *in vivo* material parameters.

Patient no.	Stress from M1 (kPa)	Stress from M2 (kPa)	Stress from M3 (kPa)	Stress RE for M2 (%)	Stress RE for M3 (%)
AD1	392.09	392.09	395.27	0.00	0.81
AD2	606.76	545.41	540.91	−10.11	−10.85
AD3	530.88	551.68	548.11	3.92	3.25
AD4	456.02	476.72	470.54	4.54	3.18
AD5	160.71	173.08	171.10	7.70	6.47
N1	272.67	243.54	240.97	−10.68	−11.63
N2	310.33	342.42	341.06	10.34	9.90
N3	218.48	248.70	246.80	13.84	12.97
N4	509.89	487.20	486.31	−4.45	−4.63
N5	252.22	256.25	252.44	1.60	0.09

RE, relative error.

## 4 Discussion

The mechanical behavior of aortic tissues is influenced by hemodynamic forces, extracellular matrix composition (particularly the microstructure of collagen and elastin networks), and endothelium–smooth muscle cell interactions. Under the physiological conditions, blood vessels exhibit nonlinear viscoelastic and anisotropic behaviors and are subjected to pulsatile pressure. Conventional quasi-static *in vitro* testing fails to fully capture these complex material behaviors, thus limiting the accuracy of extrapolated physiological biomechanical parameters. Thus, it is of paramount importance to determine the material properties of blood vessels (especially the aorta) *in vivo*, primarily because it enables the acquisition of individualized biomechanical parameters. The subject-specific *in vivo* aortic material properties not only have a profound impact on biomechanics but also have a key application value in clinical diagnosis, disease treatment, and medical device development.

In this study, a non-invasive approach for evaluating *in vivo* material properties was employed to assess the material properties of normal and pathological human ascending aortic tissues. This *in vivo* approach considers aortic geometry, non-invasive pressure condition, and the material anisotropy of aortic tissues. *In vivo* material properties determined using the non-invasive approach were compared with *ex vivo* material properties obtained using biaxial tensile testing. The effect of the initial guesses of material parameters to determine *in vivo* material properties was analyzed. This study may be considered a proof-of-concept investigation of the proposed iterative algorithm. Our primary goal was to assess the feasibility of the non-invasive method for evaluating the material properties of ascending aortic tissue under normal and pathological conditions.

### 4.1 *In vivo* and *ex vivo* material properties of ascending aortic tissues

In clinical practice, pre-operative characterization of patient-specific biomechanical properties of ascending aortic tissues remains inherently challenging. To eliminate the need for *ex vivo* testing, inverse approaches have been developed to evaluate the aortic material properties with the assistance of medical imaging for normal and pathological human ascending aortic tissues (Trabelsi et al., 2015; Liu et al., 2017; Cosentino et al., 2019). Since the stress–strain response of arteries is hyper-elastic, highly nonlinear, incompressible, and anisotropic, inverse estimation of their material properties is a complex multi-parameter nonlinear inverse problem. Trabelsi et al. (2015) suggested a non-invasive approach to identify material parameters using *in vivo* gated CT and compared the mechanical properties of five samples of ascending thoracic aortic aneurysm using their proposed *in vivo* approach and *ex vivo* testing. Under the assumption that material is isotropic, their results indicated that stiffness values measured *ex vivo* was higher than those measured *in vivo* in three patients and stiffness values measured *ex vivo* was lower than those measured *in vivo* in the remaining two patients. To address the difficulty in obtaining patient-specific material properties, Liu et al. (2017) developed a new inverse method based on aortic wall stress computation and validated the accuracy of their proposed method using the *in vivo* data from four patients with ascending aortic aneurysm. These *in vivo* material properties of four patients determined using their proposed inverse method were comparable to the experimental material properties. Cosentino et al. (2019) employed an optimization algorithm to identify *in vivo* material parameters based on the deformation relationship between the two loading states and the constitutive model with an initial guess of material parameters. Both the anisotropic Fung-type model and the isotropic Yeoh model were used to evaluate the performance of their optimization algorithm. Based on their obtained stress–strain curves, the maximum RE in stress at the maximum value of peak systolic strain reached approximately 50% between the *in vivo* and *ex vivo* methods. *In vivo* anisotropic material properties of 10 subjects were obtained using our proposed non-invasive method and compared with the *ex vivo* material properties. Overall, our results indicated that the *in vivo* iterative algorithm yielded softer material properties than the *ex vivo* aortic tissue in the circumferential direction (Tables 3–5). Except for AD3 and AD4, YMa values of the *in vivo* material properties were also lower than those of the *ex vivo* material. Consequently, vascular tissue properties obtained using the *in vivo* method exhibit lower stiffness values than those calculated using *ex vivo* measurements.

### 4.2 Effect of the initial guess of material parameters on the determined *in vivo* material properties

Three settings of initial material parameters were used to investigate their effects on *in vivo* properties determined using the proposed non-invasive method. In the first setting (M_01_), using *ex vivo* material properties as the initial guess can approximately estimate *in vivo* mechanical properties for each patient (e.g., similar anisotropy and comparable orders of magnitude of stiffness for *ex vivo* and *in vivo* estimates) with a rapid convergence rate (our triple-loop algorithm achieved inner loop convergence using M_01_). In a real-world scenario, patient-specific aortic *ex vivo* material parameters are not available before aortic replacement surgery. To improve the practical applicability of the proposed *in vivo* method for patient monitoring before surgery, a second setting was used with the same initial material parameters for all 10 subjects (M_02_). In the third setting, all 10 subjects were also assigned the same initial parameters (M_03_) to analyze how the initial parameter variations with a 5% change would affect the derived *in vivo* properties. To the best of our knowledge, this type of parameter sensitivity analysis has not been previously documented in the literature. Our results indicated that in the circumferential direction, three different initial guesses of material parameters did not exert significant effects on the determined *in vivo* material properties. The largest difference in the obtained *in vivo* YMc values was 32.7% among three initial settings across the 10 subjects. In the axial direction, the difference was relatively larger. If patient AD4 was not considered, the largest difference in obtained *in vivo* YMa values was 41.6% between the M_01_ and M_02_ initial settings for the remaining nine cases. For patient AD4, the large difference in the YMa values could be attributed to the large difference in the material anisotropy of aortic tissue represented by two settings (M_01_ and M_02_). Table 6 further confirms that the initial guess of material parameters could affect YMa values of anisotropic tissues. The closer the anisotropy of the initial material parameters is to that of the actual aortic tissue, the more accurate and computationally efficient the material property estimates obtained by the iterative algorithm are. A comparison of Tables 4 and 5 reveals that a 5% variation in the initial material parameters induced changes of 3.67% ± 2.62% and 2.79% ± 1.48% in the *in vivo* measurements of YMc and YMa, respectively.

### 4.3 Limitations

(1) One limitation of this study is the assumption of material homogeneity in the circumferential direction. In the real-world scenario, the aortic wall exhibits regional heterogeneity in mechanical properties circumferentially (Cosentino et al., 2023). Due to the limited data available from the current image data, it is deemed impractical to estimate the aortic material properties with regional heterogeneity along the circumferential direction. To address this, we assumed uniform anisotropic material properties for the aortic tissue along the circumferential direction in our model. Notably, if more detailed information on local deformation or movement of the aortic tissues could be obtained using some advanced imaging technologies (such as speckle tracking echocardiography to record local myocardial deformation), aortic material properties with regional heterogeneity might also be estimated. (2) The finite element model used in our study was the solid model and not the fluid–structure interaction (FSI) model. Hemodynamic factors should be considered in future studies while using FSI models. (3) The axial shrinkage was kept constant in our model. Currently, there is no methodology to determine the *in vivo* axial shrinkage ratio of aorta in medical images. (4) In clinical practice, neither TEE nor CT images are required for tissue donation. (Sometimes, this is impractical because some organ donors may have died from severe injuries before any TEE and/or CT imaging could be performed.) Therefore, TEE/CT images from five matched volunteers with no aortic diseases were used as substitutes for these five organ donors. It is worth noting that inherent physiological differences exist between organ donors, from whom the aortic tissue is acquired, and healthy volunteers, from whom imaging data are extracted, which may influence the results. To minimize the difference, efforts have been made to select volunteers with clinical information, including age, gender, and pressure conditions, to match that of the organ donors when pairing subjects. (5) Finally, a small sample size was used in our study. However, the acquisition of human aortic tissue samples, particularly the normal ones from organ donors, presents considerable challenges due to the limited number of organ donors and small tissue sample size. For patient data, obtaining the aortic tissue sample, CT/TEE image data, and blood pressure data from one specific patient remains challenging. All these limitations and practical constraints led us to recruit five samples in each group for this study. Nevertheless, we acknowledge that large-scale studies with a large sample size are warranted for further validation with stronger statistical power.

## 5 Conclusion

A non-invasive method was proposed for estimating subject-specific anisotropic constitutive parameters of the aortic wall using two *in vivo* aortic geometries at systolic and diastolic pressures. The finite element model-based iterative algorithm was performed on normal and pathological human ascending aortas with different degrees of anisotropy, and the results were compared with experimental material properties obtained using biaxial tensile testing. Overall, *in vivo* material properties estimated using our proposed non-invasive method exhibited lower YMc values than *ex vivo* material properties for normal and dissected ascending aortas. The proposed method could be easily extended to estimate *in vivo* material properties of other anisotropic biomaterials when relevant clinical data are available.

## Data Availability

The raw data supporting the conclusions of this article will be made available by the authors, without undue reservation.
