# Supplementary material for: Non-invasive estimation of material properties of normal and dissected human ascending aortas in vivo: comparison with the ex vivo tensile experiment
Source: Front Bioeng Biotechnol. 2026 Jan 8;13:1689692. doi: 10.3389/fbioe.2025.1689692 (PMC12824021; doi:10.3389/fbioe.2025.1689692)
Supplement: Supplementary file 1 [file Supplementaryfile1.docx]

**Supplementary Material**

**Section A: Sample thickness and model thickness**

The outer-boundary of the aorta is often adhered to surrounding tissues. Since these adherent tissues exhibit similar radiodensity to the aortic wall on CT images, accurately identifying the contour of the aortic outer boundary is challenging. Therefore, the wall thickness derived from CT-based aortic contour segmentation is generally overestimated compared to the true anatomical thickness, particularly at sites of adhesion. Therefore, the measured thickness of the ex vivo aortic specimen was employed as a threshold to constrain the wall thickness in our thin-slice models. Specifically, at any point where the segmented aortic wall thickness exceeded this threshold, the initial outer boundary contour (derived from CT segmentation) was corrected by setting the local thickness to the measured ex vivo value. The final corrected contour was used to construct a thin-slice model.

References:

Guo, X., Gong, C., Zhai, Y., Yu, H., Li, J., Sun, H., et al. (2023). Biomechanical characterization of normal and pathological human ascending aortic tissues via biaxial testing Experiment, constitutive modeling and finite element analysis. *Computers in Biology and Medicine* 166**,** 107561. doi: 10.1016/j.compbiomed.2023.107561.

**Section B: Curve fitting**


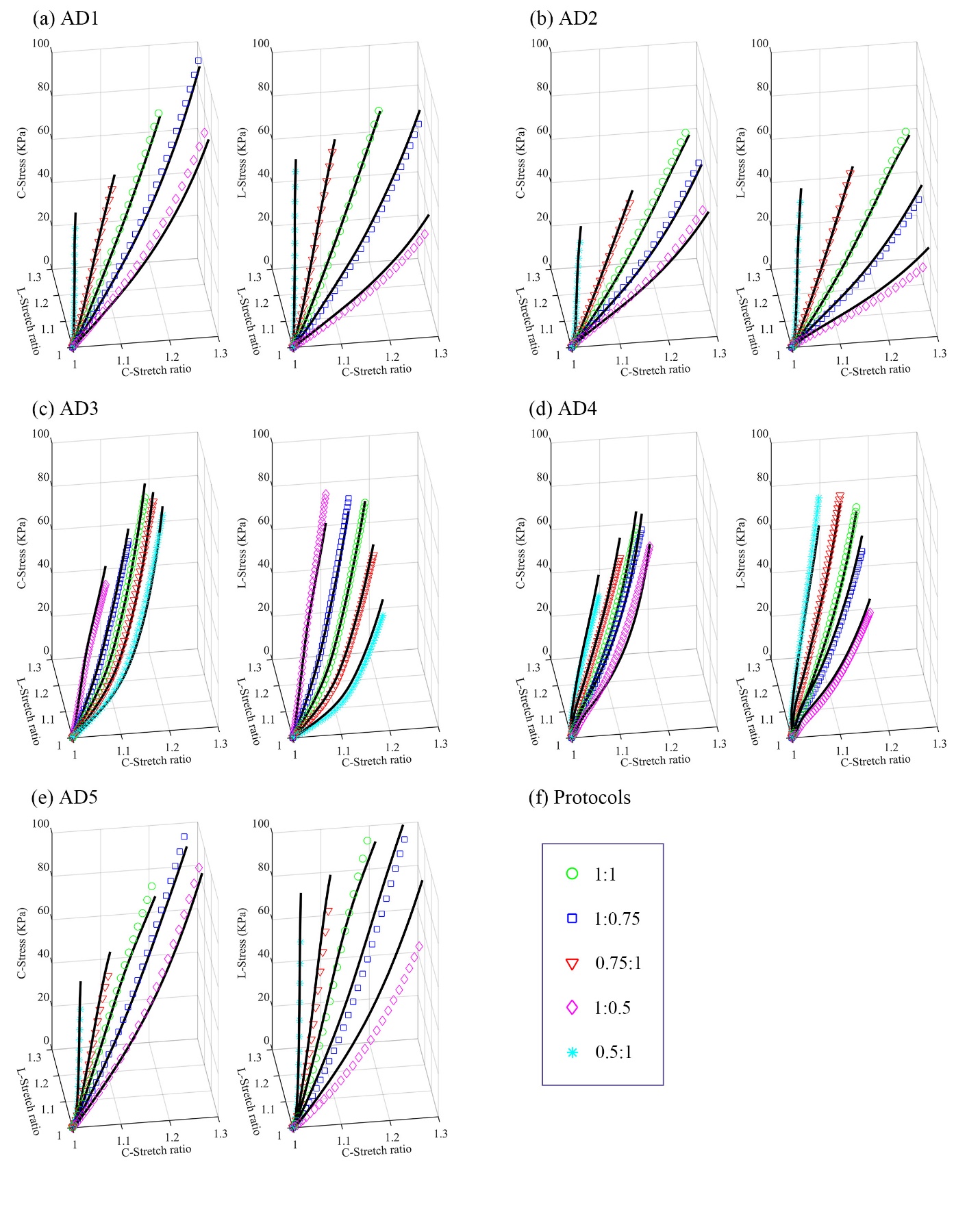


**Figure S1.** Biaxial mechanical stress-stretch ratio data and fitted material curves of five patients with AD. Ex vivo data corresponding to the five distinct protocols are denoted by different colors.


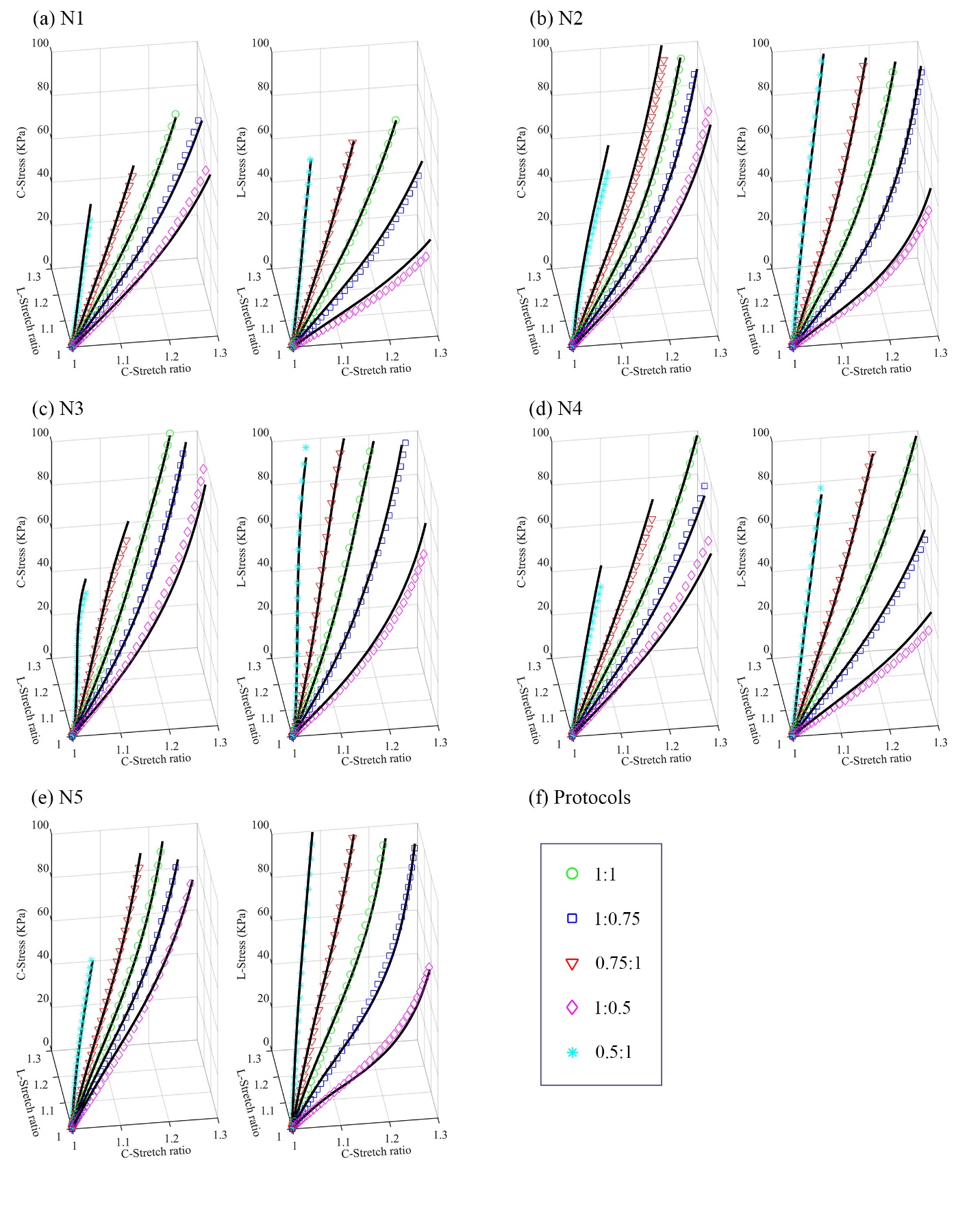


**Figure S2.** Biaxial mechanical stress-stretch ratio data and fitted material curves of five donors with normal aorta. Ex vivo data corresponding to the five distinct protocols are denoted by different colors.

**Section C: Histological analysis:**

Histological analysis of aortic tissues was performed following our previously established methodologies (Guo et al., 2023; Guo et al., 2024). Consecutive sections were stained with Oil Red O for lipid and hematoxylin–eosin (HE) for gross aortic wall morphology, respectively. Figure S3(B) shows an Oil Red O staining, where the red areas indicate lipid accumulation, suggesting the presence of atherosclerosis in AD4. Figure S3(A) shows more disorganized tissue fiber orientation, compared to other AD tissue sample and normal tissue sample.


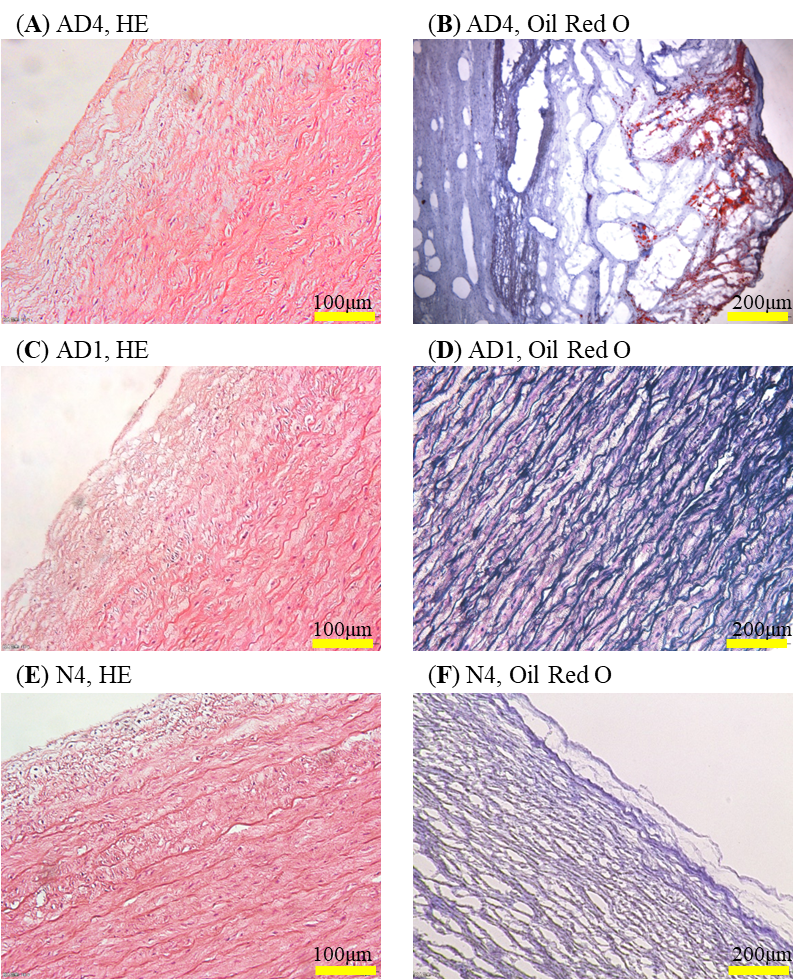


**Figure S3.** Histological sections of aortic samples cut from AD4, AD1 and N4 aorta specimens, respectively.

References:

Guo, X., Gong, C., Zhai, Y., Yu, H., Li, J., Sun, H., et al. (2023). Biomechanical characterization of normal and pathological human ascending aortic tissues via biaxial testing Experiment, constitutive modeling and finite element analysis. *Computers in Biology and Medicine* 166**,** 107561. doi: 10.1016/j.compbiomed.2023.107561.

Guo, X., Yu, H., Wang, L., Zhai, Y., Li, J., Tang, D., et al. (2024). Layer-specific biomechanical and histological properties of normal and dissected human ascending aortas. *Heliyon* 10(14)**,** e34646. doi: 10.1016/j.heliyon.2024.e34646.
